# Characterizing the Mechanical Stiffness of Passive-Dynamic Ankle-Foot Orthosis Struts

**DOI:** 10.3389/fresc.2022.820285

**Published:** 2022-04-15

**Authors:** Kara R. Ashcraft, Alena M. Grabowski

**Affiliations:** ^1^Applied Biomechanics Lab, Department of Integrative Physiology, University of Colorado Boulder, Boulder, CO, United States; ^2^Applied Biomechanics Lab, Department of Veterans Affairs, Eastern Colorado Healthcare System, Denver, CO, United States

**Keywords:** orthotic, IDEO, limb salvage, ankle stiffness, running, biomechanics

## Abstract

People with lower limb impairment can participate in activities such as running with the use of a passive-dynamic ankle-foot orthosis (PD-AFO). Specifically, the Intrepid Dynamic Exoskeletal Orthosis (IDEO) is a PD-AFO design that includes a carbon-fiber strut, which attaches posteriorly to a custom-fabricated tibial cuff and foot plate and acts in parallel with the impaired biological ankle joint to control sagittal and mediolateral motion, while allowing elastic energy storage and return during the stance phase of running. The strut stiffness affects the extent to which the orthosis keeps the impaired biological ankle in a neutral position by controling sagittal and mediolateral motion. The struts are currently manufactured to a thickness that corresponds with one of five stiffness categories (1 = least stiff, 5 = most stiff) and are prescribed to patients based on their body mass and activity level. However, the stiffness values of IDEO carbon-fiber struts have not been systematically determined, and these values can inform dynamic function and biomimetic PD-AFO prescription and design. The PD-AFO strut primarily deflects in the anterior direction (ankle dorsiflexion), and resists deflection in the posterior direction (ankle plantarflexion) during the stance phase of running. Thus, we constructed a custom apparatus and measured strut stiffness for 0.18 radians (10°) of anterior deflection and 0.09 radians (5°) of posterior deflection. We measured the applied moment and strut deflection to compute angular stiffness, the quotient of moment and angle. The strut moment-angle curves for anterior and posterior deflection were well characterized by a linear relationship. The strut stiffness values for categories 1–5 at 0.18 radians (10°) of anterior deflection were 0.73–1.74 kN·m/rad and at 0.09 radians (5°) of posterior deflection were 0.86–2.73 kN·m/rad. Since a PD-AFO strut acts in parallel with the impaired biological ankle, the strut and impaired biological ankle angular stiffness sum to equal total stiffness. Thus, strut stiffness directly affects total ankle joint stiffness, which in turn affects ankle motion and energy storage and return during running. Future research is planned to better understand how use of a running-specific PD-AFO with different strut stiffness affects the biomechanics and metabolic costs of running in people with lower limb impairment.

## Introduction

The use of a passive-dynamic ankle-foot orthosis (PD-AFO) enables people with lower limb impairment to participate in dynamic high-impact activities such as running. The Intrepid Dynamic Exoskeletal Orthosis (IDEO) is a PD-AFO specifically designed to enable people with lower limb impairment to return to running (1). The IDEO is primarily comprised of carbon fiber, which allows elastic energy storage and return during the stance phase of running (2). Use of the IDEO has improved performance and comfort in people with lower limb impairment during agility, power, and speed tests compared to use of a conventional plastic AFO (2). An IDEO consists of a custom fabricated carbon fiber foot plate and tibial cuff that are attached by a carbon-fiber strut, which is located posterior to and in parallel with the impaired biological ankle joint. The stiffness of the carbon-fiber strut determines the extent to which the IDEO keeps the biological ankle in a neutral position and controls sagittal and mediolateral motion, which can mitigate pain and prevent further injury to the impaired biological ankle joint during running. An IDEO strut is prescribed to patients using a manufacturer recommended stiffness category based on the user's mass and the amount of impaired ankle support needed (1), where a higher numerical stiffness category is recommended for people with greater body mass and/or who need greater control of impaired ankle range of motion (1). The strut acts in parallel with the impaired biological ankle, where the angular stiffness of the carbon-fiber strut determines the extent of elastic energy storage and return during the ground contact phase of running (3) and affects overall running biomechanics such as ankle joint kinematics and kinetics and leg stiffness, which could ultimately affect the metabolic cost of running, as well as lower limb agility, power production, and running speed of people with lower limb impairment (2–6). The carbon fiber footplate within the IDEO maintains the ankle in a plantarflexed position of ~0.12 rad (7°) (1), and the strut stiffness acts to control ankle dorsiflexion and plantarflexion within a smaller range of motion than that of an unimpaired biological ankle (1). Additionally, due to the plantarflexed position of the footplate, people with lower limb impairment are encouraged to utilize a midfoot or forefoot strike pattern during the stance phase of running to maximize the energy storage and return of the IDEO strut (7). However, previous studies have not independently measured angular strut stiffness. Thus, we sought to quantify IDEO strut stiffness (in kN·m/rad) to better inform prescription and characterize the biomechanics of PD-AFO use during running.

Because an IDEO strut acts in parallel with the impaired biological ankle joint, total angular ankle joint stiffness equals the sum of the strut stiffness and impaired biological ankle joint stiffness. However, when people with a lower limb impairment use a more-stiff PD-AFO, they may adjust their knee and hip joint mechanics during running, which could indirectly affect their total ankle joint stiffness through changes in ankle angle or moment during running. Esposito et al. (6) measured total ankle joint stiffness in people with lower limb impairment using a PD-AFO with three different posterior struts with a nomial stiffness, and that were 20% more stiff and 20% less stiff than nominal, during running at 3.55 m/s. The struts were made of Nylon 11 and were laser-sintered to be a specific thickness to achieve these stiffness values. They found that a PD-AFO with a strut that was 40% more stiff (992 kN/m) than the most compliant strut (667 kN/m), resulted in an approximately 18% increase in total ankle joint stiffness from 1.5 to 1.7 kN·m/rad, on average. In this same study, the unimpaired biological ankle joint stiffness was near constant regardless of the PD-AFO stiffness and 150% lower on average than the total impaired ankle joint stiffness including the PD-AFO.

A person with a lower limb impairment requires control of sagittal and mediolateral motion in the impaired biological ankle joint to decrease pain during running (2), which can be achieved through use of a PD-AFO with an appropriate strut stiffness. Yet, if all else is held constant, greater total ankle joint stiffness may increase the risk of additional injuries such as knee joint osteoarthritis or lower limb stress fractures due to greater loading rates and lower ground reaction force impact attenuation in the impaired leg and increased biomechanical asymmetry between legs (6, 8, 9). Thus, the PD-AFO strut stiffness affects overall running biomechanics, and there likely exists an optimal strut stiffness that provides control of ankle joint range of motion without increasing the risk of additional injury during running in people with lower limb impairment.

Previous studies have shown that unimpaired biological ankle joint stiffness is best characterized by a curvilinear moment-angle relationship during running at moderate speeds (2.6–3.8 m/s), where the ankle stiffens as it undergoes dorsiflexion during the first half of the stance phase (10). Moreover, a previous study that applied a compression force on an entire IDEO PD-AFO found that the force-displacement profile (in N/mm) was curvilinear (6). If the IDEO strut stiffness is curvilinear, then its stiffness depends on the magnitude and position of the applied ground reaction force during the stance phase of running. Additionally, because the unimpaired biological ankle joint is well-characterized by angular stiffness (in kN·m/rad) during running (11), the IDEO strut stiffness, which acts in parallel with the impaired biological ankle joint, should be characterized by a moment-angle relationship to understand its dynamic function (6). Thus, we characterized the strut moment-angle relationship for the range of commercially available strut stiffness categories of an IDEO PD-AFO. We predicted that each strut stiffness category (categories 1–5) would have a distinct stiffness value and that stiffness would change by the same magnitude between categories due to the manufacturer recommendations based on body mass (“PDE Individual 250 mm Springs.” Fabtech Systems)^1^. Moreover, based on previous findings that the entire IDEO PD-AFO exhibits a curvilinear force-displacement relationship (6), we hypothesized that the PD-AFO strut moment-angle curves would be better characterized by a curvilinear compared to linear relationship.

## Materials and Methods

**Figure 1:**
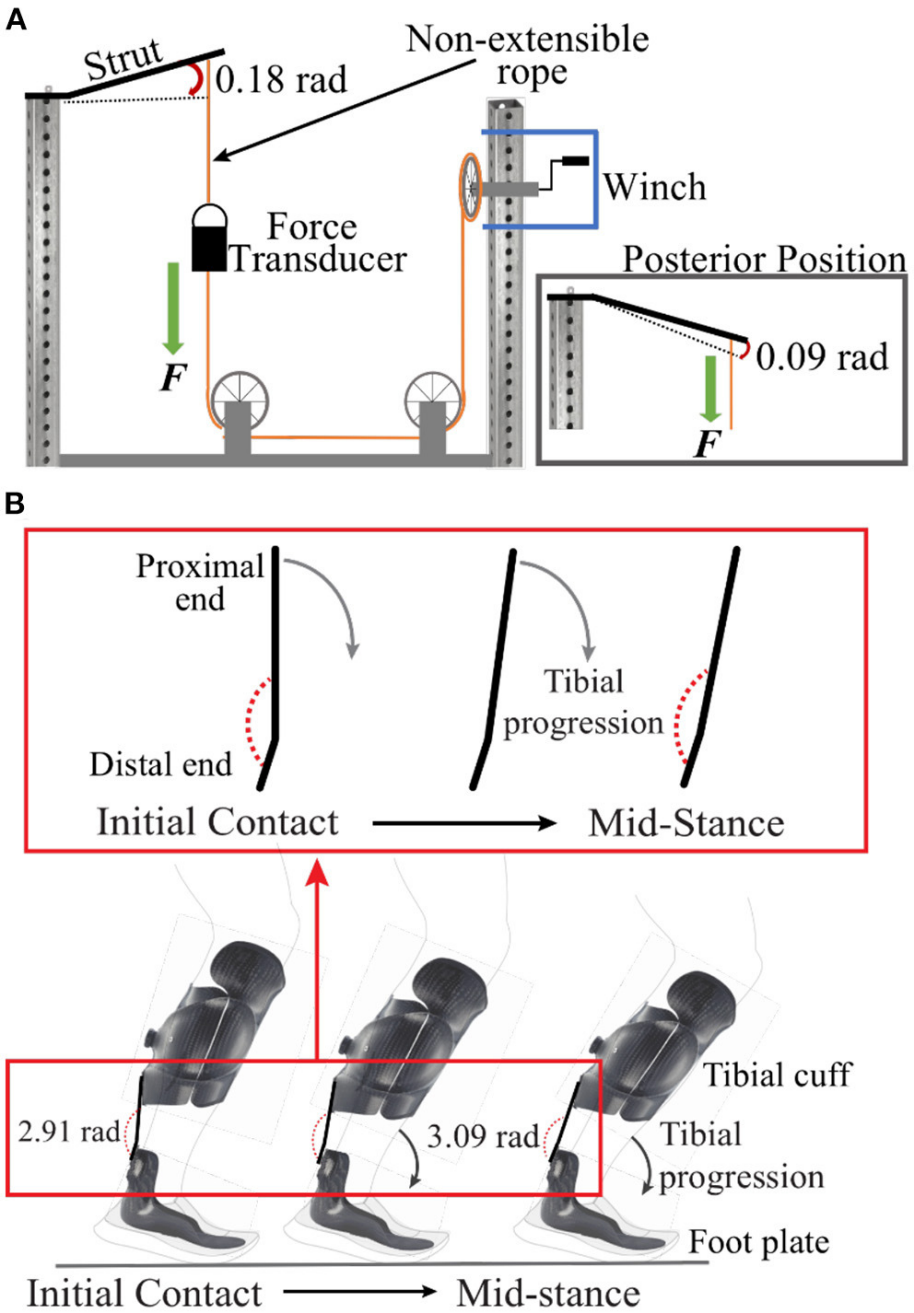
**(A)** Illustration of the custom bending apparatus. During testing, the distal end of each IDEO strut was rigidly secured to a steel frame and force was applied to the proximal end of the strut using a non-extensible rope and hand cranked winch. A force transducer was placed in-line with the non-extensible rope. We applied force to deflect the strut by 0.18 rad (10°) in the anterior direction (pictured) and 0.09 rad (5°) in the posterior direction by re-attaching the distal end of the strut to the frame (inset). For posterior deflection, we removed the strut and flipped it so that the rope was attached to the opposite surface and was parallel to the attachment point for anterior deflection. **(B)** Illustration of the anterior deflection (0.18 rad) of the IDEO strut during the stance phase of running, from initial contact to mid-stance. When no force is applied, the angle between the proximal and distal end of the IDEO strut is 2.89 rad (165°). As the tibial cuff rotates over the footplate during the first half of stance, the proximal end of the strut and tibial cuff rotate forward and the angle between the proximal and distal end of the strut increases by ~0.18 rad (10°).

We determined IDEO strut stiffness from strut stiffness categories 1, 2, 3, 4, and 5 (Fabtech, Everett, WA) using a custom bending apparatus that applied force to deflect the strut in the anterior and posterior directions relative to the user (Figure 1A). We defined anterior deflection as the proximal portion of the strut rotating in the anterior direction relative to the user during the stance phase, and measured the magnitude of the deflection relative to the distal end of the strut (Figure 1B). We defined posterior deflection as the proximal end of the strut rotating in the posterior direction relative to the user during the stance phase, and measured the magnitude of the deflection relative to the distal end of the strut. We applied force to the proximal end of the strut to reach a maximum deflection of 0.18 rad (10°) in the anterior direction, and 0.09 rad (5°) in the posterior direction (Figure 1A). We chose a maximum anterior deflection angle based on a previous study of people with lower limb impairment using a PD-AFO of manufacturer-recommended stiffness that found that the impaired ankle joint including the PD-AFO dorsiflexes (anterior deflection) by ~0.18 rad (10°) during the first half of the stance phase during running at 3.55–3.74 m/s (6). We arbitrarily chose the maximum posterior deflection angle to determine the sagittal plane stiffness provided by the strut, which affects range of motion, as the IDEO is designed to allow <0.12 rad (7°) of plantarflexion during running (1).

To determine strut stiffness, we rigidly secured the distal end of each strut to a steel frame using bolts and nuts and applied force to the proximal end of each strut, two struts from each stiffness category, in three successive loading and unloading cycles using a non-extensible rope and hand cranked winch (Figure 1A). The non-extensible rope was securely attached to the proximal end of the strut via a non-slip loop knot, tied through the two existing bolt holes used for attaching the strut posteriorly to the tibial cuff. We measured the applied force, which was applied 0.03 m from the proximal end of each strut, using a force transducer (1,000 Hz; Omegadyne, Stanford, CT) that was in-line with the non-extensible rope. We simultaneously measured the angular deflection of each strut using reflective markers and a motion capture system (100 Hz; Vicon Nexus, Oxford, UK). We placed reflective markers on the lateral edge of the strut 0.03 m from the distal end, 0.06 m from the distal end where the strut bends, and at the point of force application. The point of force application was 0.21 m from where the strut bends (Figure 1). We calculated the rotation of the strut about the fixed distal end and the deflection angle between the proximal and distal end of the strut using a custom MATLAB script (Mathworks, Natick, MA).

We calculated the average angular strut stiffness (*k*_*strut*_) as the slope of the moment vs. angle linear regressions in the anterior (up to 0.18 rad) and posterior (up to 0.09 rad) directions from three loading cycles of two struts from each stiffness category. We measured the change in angle of the proximal end of the strut, relative to the distal end (horizontal), and resolved the applied force to be perpendicular to the strut. We calculated the rotational moment at each time point as the product of the perpendicular force applied to the strut, which was down-sampled to 100 Hz, and the moment arm of the strut (0.21 m). We calculated the average coefficients of determination (adjusted *R*^2^) for linear and curvilinear characterizations of the moment vs. angle relationships for each loading cycle and then averaged these adjusted *R*^2^ across the three loading cycles for each category and then across categories.

We used a one-way ANOVA and *p* < 0.05 to determine if strut stiffness values were significantly different between categories for anterior and posterior deflection. We implemented a Bonferroni correction to account for multiple comparisons. We used descriptive analysis to compare the change in magnitude of the strut stiffness values between categories for anterior and posterior deflection. We then calculated and averaged the *R*^2^ values for the moment vs. angle relationships of each strut stiffness category. We used paired two-tailed *t*-tests and *p* < 0.05 to compare the average *R*^2^ values from linear and curvilinear moment vs. angle relationships across strut categories. All statistical tests were performed using R-studio (Boston, MA) software.

## Results

**Figure 2:**
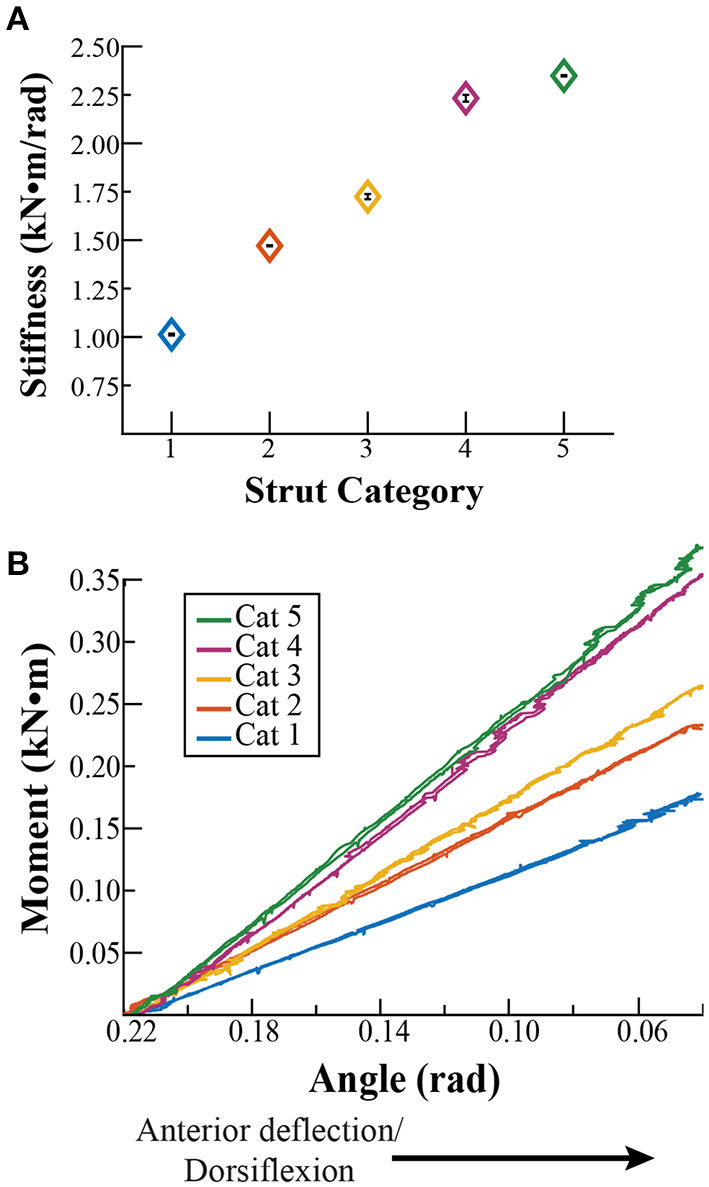
**(A)** Stiffness values (± SD) for each strut stiffness category (Cat) with anterior deflection, calculated as the quotient of the moment at an angle of 0.18 rad (10°) for Cat 1–5. **(B)** Representative moment versus angle relationships for each Cat during anterior deflection from an angle of 0.22 rad (13°) relative to horizontal at the beginning of the test, to 0.04 (2.3°) rad at the finishing point of the test, for 0.18 rad (10°)of deflection.

With anterior deflection, the average ± SD stiffness values for strut stiffness categories 1–5 were 1.01 ± 0.01, 1.47 ± 0.01, 1.72 ± 0.02, 2.23 ± 0.01, and 2.35 ± 0.02 kN·m/rad, respectively (Figure 2A). The stiffness value for each strut category was significantly different from the others (avg. *p* < 0.001). Contrary to our initial assumption, the stiffness values did not change by the same magnitude between categories with anterior deflection. Stiffness increased by 0.46, 0.25, 0.51, and 0.12 kN·m/rad between categories 1 and 2, 2 and 3, 3 and 4, and 4 and 5, respectively. We found no significant difference between the adjusted *R*^2^ values of a 2nd degree polynomial and linear fit with anterior deflection (*p* = 0.68; Table 1). Thus, we characterized the strut moment versus angle relationship with a linear fit due to a greater numerical *R*^2^ (average linear *R*^2^ = 0.99; Figure 2B and Table 2).

**Table 1:** Average *R*^2^ values for linear and 2nd degree polynomial curvilinear moment versus angle relationships for each strut stiffness category (Cat).

	**Cat 1**	**Cat 2**	**Cat 3**	**Cat 4**	**Cat 5**	**Average *R*^2^**
Anterior linear *R*^2^	0.999	0.999	0.999	0.999	0.999	0.999
Anterior curvilinear *R*^2^	0.999	0.999	0.999	0.999	0.999	0.999
Posterior linear *R*^2^	0.996	0.998	0.992	0.991	0.996	0.995
Posterior curvilinear *R*^2^	0.996	0.998	0.996	0.995	0.997	0.996

**Table 2:** Average linear regression equations for each category for both the anterior and posterior directions.

	**Cat 1**	**Cat 2**	**Cat 3**	**Cat 4**	**Cat 5**
Anterior linear equation	τ = 1.01·θ −1.1e^−3^	τ = 1.47·θ-3.0e^−4^	τ = 1.73·θ-1.9e^−3^	τ = 2.62·θ-5.3e^−3^	τ = 2.36·θ-2.1e^−3^
Posterior linear equation	τ = 0.84·θ −4.3e^−4^	τ = 1.44·θ −1.3e^−3^	τ = 1.58·θ −8.0e^−4^	τ = 2.00·θ −8.0e^−4^	τ = 2.76·θ −1.0e^−4^

*The equations indicate the angle of rotation of the proximal strut (θ) in radians, used to calculate the strut moment (τ) in kN·m. Average angular strut stiffness (k_strut_) is the slope of the moment versus angle linear regression*.

**Figure 3:**
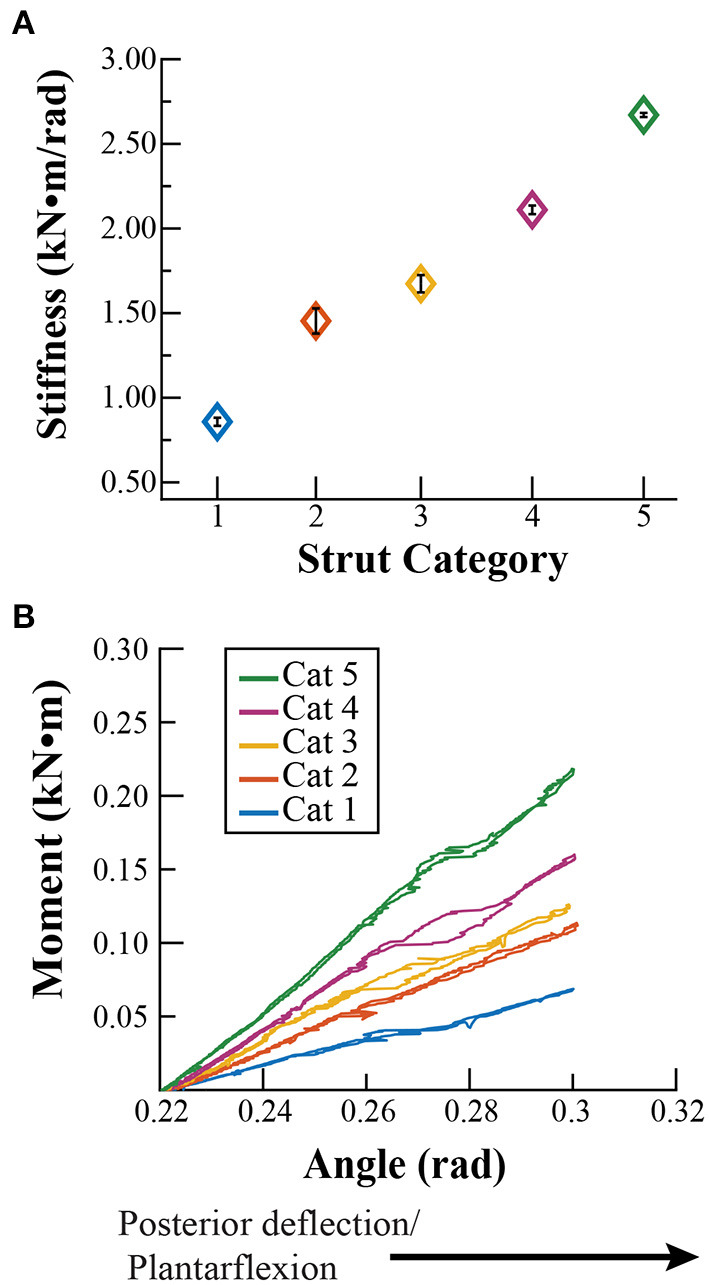
**(A)** Stiffness values (± SD) for each strut stiffness category (Cat) with posterior deflection, calculated as the quotient of the rotational moment at an angle of 0.09 rad (5°) for Cat 1–5. **(B)** Representative moment versus angle relationships for each Cat during posterior deflection from an angle of −0.22 rad (−13°) relative to horizontal at the beginning of the test, to −0.32 (−18°) rad at the finishing point of the test, for 0.09 rad (5°) of deflection.

With posterior deflection, the average ± SD stiffness values for strut stiffness categories 1–5 were 0.84 ± 0.02, 1.43 ± 0.07, 1.57 ± 0.05, 2.01 ± 0.03, and 2.76 ± 0.01 kN·m/rad, respectively (Figure 3A). The stiffness value for each strut category was significantly different from the others (avg. *p* < 0.001), with the exception of categories 2 and 3, where we detected no significant difference between stiffness values (*p* = 0.07). Contrary to our initial assumption, the stiffness values did not change by the same magnitude between categories with posterior deflection. Stiffness increased by 0.59, 0.14, 0.44, and 0.75 kN·m/rad between categories 1 and 2, 2 and 3, 3 and 4, and 4 and 5, respectively. We found no significant difference between the adjusted *R*^2^ values of a 2nd degree polynomial and linear fit with posterior deflection (*p* = 0.10). Thus, we characterized the strut moment versus angle relationship with a linear fit due to a greater numerical *R*^2^ (average linear *R*^2^ = 0.99; Figure 3B and Table 2).

## Discussion

Our initial assumption was partially supported. For anterior and posterior deflection, all of the stiffness values for each strut stiffness category were different from each other, apart from categories 2 and 3 for posterior deflection. In addition, the stiffness values for each strut stiffness category changed by different magnitudes between categories for anterior and posterior deflection. The lack of difference in stiffness values between some of the strut stiffness categories and the different changes in stiffness values between categories could be due to the manufacturing process of carbon fiber struts where fabrication is done by hand. For example, there was no difference in the stiffness value between strut categories 2 and 3 for posterior deflection, which may indicate that strut fabrication is variable. Thus, it is imperative for manufacturers to test and quantify the actual stiffness values of each PD-AFO strut prior to prescription.

We reject our hypothesis that strut angular stiffness is better characterized by a curvilinear relationship for anterior and posterior deflection because we found no difference between curvilinear and linear moment versus angle relationships. During running at 3.8 m/s, the angular stiffness of an unimpaired biological ankle is ~1.2 kN·m/rad at 0.18 rad (10°) of dorsiflexion and has a curvilinear moment versus angle relationship, where the ankle becomes stiffer with greater dorsiflexion (4, 10). Thus, based on our findings, if no other joint-level adjustments are made, use of a PD-AFO with a strut stiffness category 3 (manufacturer recommended stiffness category for a 70 kg person), which has a stiffness of 1.72 kN·m/rad in the anterior direction, combined with an impaired biological ankle would more than double total ankle joint stiffness during running. Wach et al. (12) characterized the compressive stiffness of an entire IDEO PD-AFO in N/mm by simulating discrete time-points of the stance phase of walking and found that the combined stiffness of the IDEO PD-AFO plus a surrogate limb was most stiff at mid-stance, when the ankle dorsiflexes and least stiff at pre-swing, when the ankle plantarflexes (95 and 45 N/mm, respectively), which is similar to the angular stiffness of an unimpaired biological ankle during the stance phase of running. However, Wach et al. measured the sagittal plane rotation of the proximal end of the IDEO PD-AFO from 0.04 rad (2.3°) during loading at mid-stance to 0.05 rad (2.9°) during loading at pre-swing, suggesting that the compressive stiffness does not fully describe the mechanical behavior or function of the PD-AFO during locomotion (6). Additional research is required to better understand how the PD-AFO strut deforms during loading as well as how it interacts with the injured ankle, foot plate and tibial cuff, as these elements likely affect the user's biomechanics during running.

In addition to controlling sagittal and mediolateral ankle joint range of motion, the stiffness profile of a PD-AFO strut affects leg stiffness and elastic energy storage and return during the stance phase of running. The stance phase of running is well-characterized by a spring-mass model, where leg stiffness equals the quotient of peak ground reaction force and total leg displacement (13), and primarily depends on ankle joint stiffness during hopping and running (14, 15). However, to the best of our knowledge, the effects of springs with different stiffness profiles that act in parallel with the ankle are unknown. The spring stiffness profile refers to the slope of the force versus displacement or moment versus angle relationship. The stiffness profile of a spring within a passive-elastic exoskeleton in parallel with the legs affects elastic energy storage and return and metabolic cost during hopping (16). Specifically, use of this passive-elastic exoskeleton that had springs with a curvilinear stiffness profile, where stiffness decreased with compression (degressive), resulted in the greatest elastic energy return and lowest metabolic cost compared to springs with a linear stiffness profile and with a curvilinear stiffness profile where stiffness increased with compression. Moreover, use of the exoskeleton with degressive stiffness springs reduced metabolic cost by 13–24% compared to hopping without an exoskeleton over a range of frequencies (16). Thus, use of a PD-AFO with a degressive strut stiffness profile, rather than a linear stiffness profile, may allow the user to run with a lower metabolic cost, which could ultimately improve their performance (17). Future studies are warranted to understand the effects of different PD-AFO strut stiffness profiles on running biomechanics and metabolic costs.

PD-AFO strut stiffness directly affects total ankle joint stiffness, which in turn affects ankle range of motion, energy storage and total leg stiffness during running. Hewett et al. (18) found that runners with increased leg stiffness had higher loading rates during the stance phase of running, and a higher incidence of stress fractures due to reduced ground reaction force impact attenuation, compared to runners with lower leg stiffness (8, 18). However, it has also been reported that runners with lower leg stiffness expend more metabolic energy during running, and have a higher incidence of soft tissue injury, such as ligament damage, due to greater joint motion (19, 20). Thus, there likely exists an optimal leg stiffness during running that mitigates injury risk while improving performance through reductions in metabolic cost. Moreover, unimpaired biological legs can adjust ankle, knee and hip joint stiffness to maintain leg stiffness during running across different speeds or terrain (21), however it is unknown whether an increase in total joint stiffness for the impaired biological ankle of runners using a PD-AFO results in changes to knee joint stiffness and/or leg stiffness. Moreover, future studies are needed to understand if the additional stiffness provided by the PD-AFO strut results in an increase in additional injury risk for people with lower limb impairment during running.

We determined the stiffness values of carbon fiber struts that are utilized within a PD-AFO for patients with lower limb impairment. A better understanding of the stiffness values and the effects of different stiffness for PD-AFO struts can inform orthotic prescription, dynamic function, and biomimetic design. This study had several potential limitations. The purpose of this research was to quantify and characterize the stiffness of PD-AFO struts. However, when in use, the PD-AFO strut is attached to a custom-fabricated tibial cuff and foot plate (Figure 1B), which may deform during the stance phase of running, and potentially affect the overall stiffness of the PD-AFO and thus overall ankle joint stiffness. Additionally, the PD-AFO strut is designed to allow the user to change sagittal plane ankle stiffness. We chose 0.09 rad (5°) of posterior deflection for stiffness testing and this angle may not encompass the full range of motion available to the user during running. Lastly, we used PD-AFO struts that were made by a specific manufacturer, and the stiffness values provided can only be applied to this model of carbon-fiber PD-AFO struts, which may limit the generalizability of our results. We encourage manufacturers to measure and present the stiffness values of PD-AFO struts rather than arbitrary stiffness categories. Future research is planned to better understand how use of a PD-AFO with different strut stiffness, acting in parallel to the ankle, affects biomechanics and metabolic costs during dynamic activities such as running.

## Data Availability Statement

The raw data supporting the conclusions of this article will be made available by the authors, without undue reservation.

## Author Contributions

KA and AG contributed to conception and design of the study and wrote sections of the manuscript. KA acquired the data, performed the statistical analysis, and wrote the first draft of the manuscript. Both authors contributed to manuscript revision, read, and approved the submitted version.

## Funding

This work was supported by a grant from the Defense for Health Affairs, Clinical Research Intramural Initiative, CDMRPL-17-0-DMI170709.

## Author Disclaimer

The views expressed in this publication do not reflect the views or policies of the Department of the Navy, Department of Defense, Defense for Health Affairs, or United States Government.

## Conflict of Interest

The authors declare that the research was conducted in the absence of any commercial or financial relationships that could be construed as a potential conflict of interest.

## Publisher's Note

All claims expressed in this article are solely those of the authors and do not necessarily represent those of their affiliated organizations, or those of the publisher, the editors and the reviewers. Any product that may be evaluated in this article, or claim that may be made by its manufacturer, is not guaranteed or endorsed by the publisher.

## Footnotes

^1^PDE Individual 250mm Springs. Available online at: https://fabtechsystems.com/PDE-Individual-250mm-Springs.html (accessed: 11 November 2021).
